# The impact of employee level and work stress on mental health and GP service use: an analysis of a sample of Australian government employees

**DOI:** 10.1186/1471-2458-4-41

**Published:** 2004-09-30

**Authors:** Ruth A Parslow, Anthony F Jorm, Helen Christensen, Dorothy H Broom, Lyndall Strazdins, Rennie M D' Souza

**Affiliations:** 1 Centre for Mental Health Research, (CMHR) Australian National University ACTON ACT 0200 Australia; 2 National Centre for Epidemiology and Population Health (NCEPH), Australian National University ACTON ACT 0200 Australia

## Abstract

**Background:**

This study sought to identify the extent to which employee level and work stressors were associated with mental health problems experienced by Australian government employees, and with their use of primary care services.

**Methods:**

806 government employees aged between 40 and 44 years were surveyed as part of an epidemiological study conducted in Australia. Data collected from participants included sociodemographic attributes, physical health, psychological measures and work stressors relating to job control, job demands, job security and skills discretion at work. For 88% of these participants, information on visits made to general practitioners (GPs) for the six months before and after their survey interview was obtained from health insurance records.

**Results:**

When work stress and personal factors were taken into account, men at more junior levels reported better mental health, more positive affect and used fewer GP services. Women at middle-management levels obtained less GP care than their more senior counterparts. Both men and women who reported higher levels of work stress were found to have poorer mental health and well-being. The impact of such stressors on GP service use, however, differed for men and women.

**Conclusion:**

Measures of work stress and not employee level affect the mental health and well-being of government employees. For governments with responsibility for funding health care services, reducing work stress experienced by their own employees offers potential benefits by improving the health of their workforce and reducing outlays for such services.

## Background

In 1999, the World Health Organization reported that workers continued to suffer high levels of work-related injuries and deaths [1]. It also flagged, however, the increase in mental health problems reported by workers in industrialized countries as a result of their experiencing psychological stress and excessive job demands in the workplace [1]. The health consequences of such psychosocial aspects of the work environment have been examined in a range of settings across different countries. Much of this research has drawn on the model developed and refined by Karasek who proposed that work-related mental strain and associated psychiatric disorder result from combinations of, and interactions between, four different employment factors: heavy job demands, limited input to decision making processes, lack of skill discretion within the job and poor work-based social support [2,3]. Such factors, in particular those concerning decision making, skill discretion and social support have been found to be most problematic for those in lower grades of employment and to be less prevalent among employees in higher ranking positions [4-6].

The applicability of this model for the government sector is well supported by cross-sectional and longitudinal studies drawing on the Whitehall II study of a large cohort of 10,308 London-based government employees. Again, such studies have found that those in lower grades report that they have less job control, less variety in their work, and less job satisfaction [7]. Those reporting higher levels of such work stress have also been found to have greater risk of cardiovascular health problems [8] and poorer psychiatric health [5,9,10].

There has been little research undertaken on the health impact of job level and work stressors for government employees in Australia. An earlier study that explored the relationships between work stressors and blood pressure in Australian government employees, found chronic perceived work stress to be associated with blood pressure change [11]. The impact of job level and work stress on Australian government employees' mental health has not been previously explored.

We have been able to explore these issues using data collected from 806 government employees who participated in the PATH Through Life Project, a large community-based study being conducted by the Centre for Mental Health Research in Canberra, Australian Capital Territory (ACT). Survey participants provided information on sociodemographic measures, mental and physical health, employment level and work-related stress. For 88% of these participants, independently collected information on their use of general practitioner services was also available. These data have allowed us to examine the impact of employment level and work-related stress on Australian government employees' mental and physical health, their psychological well-being, and also their use of general practitioner care. We hypothesised that those working in lower level government positions would report higher levels of work-related stress, that they would be found to have more mental and physical health problems and that they would use higher numbers of primary medical services.

## Methods

### Subjects

The PATH Through Life Project is a longitudinal study of individuals living in the community with participants being drawn from three age groups: 20–24, 40–44 and 60–64 years. Those in the age group of interest for this study were aged from 40 to 44 years on 1 January 2000 and drawn from the Australian Electoral Rolls for Canberra in the Australian Capital Territory and adjacent town of Queanbeyan in New South Wales. Enrolment on these rolls is compulsory for all Australians aged 18 and over. Potential participants were drawn from a 10-year age range, the minimum range then released for research purposes by the Australian Electoral Commission. The number of potential participants found, and in the required age range, was 3919, of whom 2530 participated in the survey, giving a response rate of 64.4%.

Canberra is the national capital of Australia and many Australian Government entities are based in the ACT, including both houses of parliament, and the 16 major agencies that currently comprise the Australian Public Service. In this study, 806 respondents aged between 40 and 44 reported that they worked in office-based government administrative positions, developing and implementing government policy. As well as providing information on labour force status and the type of position held, respondents who worked in government positions were specifically asked to provide details on the level of the position they occupied. Five mutually exclusive employee categories were formed by grouping together those whose levels of employment were broadly comparable as follows. Employees who occupied positions at Australian Public Service (APS) Levels One to Four were grouped together as Junior employees and those at both the APS Levels 5 and 6 were classified as belonging to the Intermediate category of employees. Employees in the next two classifications of the APS (Executive Levels 1 and 2) were allocated to separate groups, Senior 1 and Senior 2. While those in Executive Level 1 positions develop policy and implement government programs, those at the Executive 2 level are primarily managers with direct responsibility for managing a number of employees from APS Level 1 to Executive Level 1 [12]. Finally, all respondents in the Senior Executive Service of the government were allocated to one category, Executive. The number of participants in each of these five categories of employees is given in Table 1, together with a short description of the positions included in each of those categories.

**Table 1:** Descriptions of government employee categories

Position level in Australian Public Service (APS)	Description of positions covered by these levels	Employee category	Number, % of participants		% female	Mean years of education
APS Level 1 APS Level 2 APS Level 3 APS Level 4	Work is always supervised; can include: drafting correspondence, organising travel, filing, other routine clerical work.	Junior	123	15.26	74.80	13.46
APS Level 5 APS Level 6	Work includes: supervising junior staff, liaising with external bodies, supporting project managers, drafting complex correspondence and policy papers, undertaking research.	Inter-mediate	215	26.67	51.63	14.87
APS Level 7	Work includes: managing government programs and contracts, supervising staff, preparing high level policy advice, developing and implementing government policy	Senior 1	220	27.30	37.73	15.69
APS Level 8	Work includes: managing a Section of staff, providing policy, financial, or administrative advice to government, representing department at external meetings.	Senior 2	193	23.95	33.16	16.02
Senior Executive Service	Responsible for: overall management of large numbers of staff; achieving government objectives through development and implementation of innovative and financially sound policy.	Executive	55	6.82	30.91	16.58
Total			806	100.0		
Mean measure					45.53	15.27

### Measures

Survey participants completed a questionnaire that included socio-demographic characteristics and measures of physical and mental health, and well-being. Participants in the workforce were asked 22 questions relating to their work situation. These questions matched those used in the Whitehall II study [7,13]. Nine questions related to job control, and reflected the amount of authority the worker has over decision-making [9]. Four questions concerned the manageability of job demands; the extent to which the worker is faced with difficult time and workload pressures and conflicting demands. Finally six questions addressed skill discretion and related to the variety of tasks to be done and the breadth of skills needed to undertake those tasks. For each of these 19 questions, respondents could answer: often, sometimes, rarely or never. Responses were given values of 1 to 4 with the highest score allocated to the less stressful work circumstances: those in which the individual had more job control, more manageable job demands, and higher levels of skill discretion. Participants were also asked the number of hours they usually worked per week and their assessment of their employment security and future employment opportunities. Answers to the last two questions used four point Likert-type scales and, again, were coded to give higher scores to those who reported that they had a more secure position or could obtain another job relatively easily. The mean of these two scores was used as an overall measure of job security.

Socio-demographic measures used in these analyses included sex, age, years of education, level of household responsibilities, and experience of any of six life events during the previous six months. Since each of these factors has the potential to modify an individual's mental health independently of their work stress, we adjusted for these in our final analyses. Scores for level of household responsibility were drawn from participants' responses to questions concerning the extent to which, in their household, they were responsible for four areas: household tasks, childcare, financial management, and providing money. Comparable scores for participants who did not have children in their households were then derived by calculating the mean of their measures for household tasks, financial management and providing money, and adding this to their total household responsibility score. Scores for these measures could range from zero to 16 with higher sores representing more household responsibilities.

Health measures obtained from participants and used in these analyses included: scores on Goldberg's depression and anxiety scales [14] and state measures of positive and negative affect using the Positive and Negative Affect Scales (PANAS) [15]. Measures of self-rated health, mental and physical health were taken from respondents' answers to the Medical Outcomes Study 12-item Short-Form Health Survey (SF-12) [16]. The first of these is measured by a single question in which participants rate their health as excellent, very good, good, fair or poor with higher scores indicating poorer self-rated health. Records on participants' visits to general practitioners (GPs) were also obtained. In Australia, the costs of most health care visits made to medical practitioners by Australians with citizenship or residency status are subsidised, either partly or totally, through the Australian Government funded universal health insurance scheme, Medicare. Information on the number of such visits is collected by the Health Insurance Commission. These data are used for administrative purposes and identify general practitioner and specialist services, but not the health problems addressed during each visit. While these records cover most visits made to general practitioners, they will not include a small number of services, paid for by patients but not claimed against Medicare. All participants were asked if they would consent to the researchers being provided information on the number of visits they made to general practitioners for specific periods before and after their interview. 709 (88.0%) of the 806 participants consented to this request and information on the number of GP visits they made during the six months preceding and the six months following their PATH interview was obtained from the Health Insurance Commission.

### Statistical analyses

Analyses of variance (ANOVAs) were first undertaken to examine the extent to which sociodemographic measures and work stress measures changed with level of employment. Similar analyses, conducted separately for men and women, then compared mean mental health measures across the five employee levels. Finally, regression analyses were used to examine the contribution of employee category and work attributes in explaining participants' health and health service measures, whilst controlling for the following possible modifying factors: participant's age, years of education, level of household responsibilities and life events experienced in the past six months. For these analyses, categorical variables identifying each of the five government employee categories were created and the first four of these included in the regression equations, taking the most senior category, Executive, as reference group. After initial testing indicated that two dependent variables, negative affect and use of GP services, were not normally distributed and more closely fitted the negative binomial and Poisson distributions respectively, analyses of these two measures used negative binomial and Poisson regressions respectively. Strength of associations between dependent variables and predictor variables were measured using *R*^2 ^for linear regressions and Incidence Rate Ratios when the Poisson or negative binomial regression model was used. Incidence Rate Ratios (IRRs) are interpreted in a similar manner to odds ratios and represent the expected change in the dependent variable in response to one unit change in the predictor variable. The contribution of employee level and work stress measures in explaining variation in health measures was also obtained. This contribution was measured using change in R^2 ^for linear regressions and the change in the Chi-square estimate of the fit of model for the negative binomial and the Poisson regression analyses. A final analysis examined the impact of employee category and work stressors on use of GP services, taking into account demographic, lifestyle and health measures. Analyses were undertaken using SPSS 11.5 and STATA 7 [17].

## Results

Across the five categories of government employees, there was no significant difference in education level or in numbers of life events experienced in the past six months. Employees working at higher levels, however, reported lower levels of household responsibility and had more opportunities to develop and use different skills in their work, reported more job control and felt more secure in their current jobs. As expected, those in more senior positions also had less manageable job demands and worked longer hours.

Analyses were then performed, separately for men and women, to examine differences in measures of mental health, well-being and GP service use across the five government employee categories. Level of physical health, as measured by the SF-12, was also examined for comparative purposes. The only measure to differ significantly across employee categories was level of positive affect in the past four weeks (Table 3). For both men and women, those in higher categories recorded higher scores on this measure.

**Table 3:** Mean health scores by government employee category

	Government employee category:					
	Junior	Intermediate	Senior I	Senior 2	Executive	*P*
**Men**						
Self-rated health	2.48 (2.17–2.80)	2.31 (2.14–2.48)	2.26 (2.11–2.42)	2.25 (2.10–2.40)	2.08 (1.82–2.34)	0.42
SF-12 mental health	51.91 (49.60–54.22)	50.92 (49.17– 52.66)	49.35 (47.64– 51.07)	50.52 (49.13– 51.91)	49.71 (47.50– 51.92)	0.49
SF-12 physical health	52.91 (50.91–54.92)	51.60 (50.25–52.95)	52.56 (51.36–53.76)	52.83 (51.81–53.86)	54.65 (52.47-56.83)	0.18
Goldberg Depression Score	2.50 (1.61–3.39)	2.09 (1.64–2.53)	2.17 (1.75–2.59)	2.15 (1.80–2.50)	1.45 (1.01–1.88)	0.35
Goldberg Anxiety Score	3.30 (2.27–4.33)	3.12 (2.58–3.65)	3.18 (2.69–3.66)	3.65 (3.22–4.08)	3.13 (3.22–4.08)	0.54
Positive affect	31.53 (28.89–34.18)	30.25 (28.91–31.59)	31.65 (30.46–32.84)	32.87 (31.70–34.04)	34.29 (32.74–35.84)	0.01
Negative affect	16.93 (14.98–18.89)	16.25 (14.96–17.54)	16.31 (15.34–17.29)	16.31 (15.32–17.30)	15.45 (14.00–16.90)	0.88
GP services used	2.86 (1.54–4.17)	3.24 (2.39–4.08)	3.12 (2.45–3.79)	2.14 (1.97–2.85)	2.30 (1.37–3.23)	0.33
**Women**						
Self-rated health	2.36 (2.17–2.55)	2.24 (2.09–2.39)	2.13 (1.94–2.32)	2.14 (1.90–2.38)	2.35 (1.81–2.90)	0.42
SF-12 mental health	47.98 (45.52–50.30)	48.12 (45.95–50.30)	48.40 (46.19–50.61)	51.11 (49.18–53.03)	52.60 (49.48–55.71)	0.91
SF-12 physical health	51.68 (50.12–53.24)	52.55 (51.10–53.99)	52.29 (50.58–54.01)	51.11 (49.18–53.03)	52.60 (49.48–55.74)	0.76
Goldberg Depression Score	2.76 (2.20–3.32)	2.58 (2.13–3.02)	2.35 (1.84–2.86)	2.02 (1.46–2.58)	2.24 (1.12–3.35)	0.39
Goldberg Anxiety Score	3.96 (3.37–4.54)	3.78 (3.28–4.29)	4.01 (3.39–4.63)	3.38 (2.70–4.05)	3.41 (2.21–4.62)	0.62
Positive affect	29.59 (28.14–31.03)	31.78 (30.63– 32.94)	32.05 (30.72– 33.37)	32.30 (30.68–33.91)	32.59 (30.30–34.87)	0.03
Negative affect	17.89 (16.12–19.66)	17.31 (16.05–18.57)	17.70 (16.16–19.24)	16.64 (14.76–18.52)	17.59 (14.70–20.48)	0.87
GP services used	4.21 (3.36–5.06)	5.11 (4.03–6.19)	4.47 (3.33–5.62)	3.15 (2.35–3.96)	4.73 (2.33–7.13)	0.16

We next used regression analyses to examine the impact of employee category and work stress on participants' measures of health and well-being and on their use of GP services. In preliminary testing, we found that the two measures – job control and skill discretion – both contributed significantly and independently to mental health measures, hence these measures were not combined but included separately in the analyses. The results of these analyses for men are in Table 4 and for women in Table 5. After controlling for socio-demographic and work stress measures, men in the lowest levels of employment reported significantly better mental health as measured by the SF-12 Mental Health score, higher levels of positive affect and used fewer GP services. Other measures of mental health, including self-rated health, and symptoms of anxiety and depression, did not vary significantly with employee level. Men with more manageable job demands reported better mental health, fewer depressive and anxiety symptoms and less negative affect. For men, there was a consistent association between less work stress and better health. Those with more job security or higher levels of skill discretion reported significantly better self-rated health, mental health, fewer depressive and anxiety symptoms, more positive and less negative affect and also used fewer GP services. However, those who worked fewer hours per week made more visits to GPs.

**Table 4:** Associations between health measures, and government employee category and work stressors – men

	Health measure							
Predictor variables:	Self-rated health	SF-12 Mental health	SF-12 Physical health	Goldberg Depression	Goldberg Anxiety	Positive Affect	Negative Affect	GP services obtained
	Beta^c^	Beta^c^	Beta^c^	Beta^c^	Beta^c^	Beta^c^	Incident Rate Ratio^a^	Incident Rate Ratio^a^
**Government employee category**								
Junior	-0.033	0.161*	-0.014	0.029	-0.038	0.137*	1.011	0.492***
Intermediate	-0.002	0.115	-0.183	0.096	0.021	-0.034	1.029	0.742
Senior 1	0.017	0.027	-0.151	0.141	0.040	-0.032	1.049	0.864
Senior 2	0.036	0.089	-0.120	0.112	0.078	-0.008	1.032	0.797
**Work stress measures**								
Job control	-0.060	0.031	0.019	-0.022	-0.016	0.082	0.966	0.808*
Manageable job demands	0.010	0.150**	0.037	-0.145**	-0.245***	0.019	0.879***	1.018
Usual hours per week	0.066	-0.047	-0.013	0.103	0.050	0.009	1.002	0.982***
Job security	-0.180***	0.193***	0.007	-0.171**	-0.154**	0.125**	0.942*	0.868**
Skill discretion	-0.171**	0.302***	0.024	-0.247***	-0.237***	0.398***	0.792***	0.766**
Δ*R*^2 ^*attributable to level & work stress*	0.085***	0.176***	0.016	0.134***	0.152***	0.228***	75.77^b^***	91.20^b^***

All analyses controlling for age, education, household responsibilities, and life events in past 6 months

a Incidence Rate Ratio from negative binomial or Poisson regression

b Chi-square estimate of the improvement in fit of regression model due to employee level and work stress factors.

c Standardised Beta

* p < 0.05; ** p < 0.01; *** p < 0.001

**Table 5:** Associations between health measures, and government employee category and work stressors – women

	Health measure							
Predictor variables:	Self-rated health	SF-12 Mental health	SF-12 Physical health	Goldberg Depression	Goldberg Anxiety	Positive Affect	Negative Affect	GP services obtained
	Beta^c^	Beta^c^	Beta^c^	Beta^c^	Beta^c^	Beta^c^	Incident Rate Ratio^a^	Incident Rate Ratio^a^
**Government employee category**								
Junior	-0.178	0.044	-0.015	-0.041	0.057	-0.030	0.970	0.807
Intermediate	-0.138	-0.022	0.000	0.044	0.113	0.013	1.014	1.015
Senior 1	-0.156	0.005	0.011	0.008	0.104	0.009	1.022	0.928
Senior 2	-0.073	0.066	-0.119	-0.043	0.003	0.008	0.914	0.737*
**Work stress measures**								
Job control	-0.049	0.120*	0.015	-0.099	-0.098	0.027	0.826***	0.864*
Manageable job demands	-0.123*	0.173**	0.141*	-0.245***	-0.265***	0.146*	0.852***	1.213**
Usual hours per week	0.004	-0.086	0.058	0.043	-0.023	-0.010	1.002	1.001*
Job security	-0.054	0.100	-0.076	-0.101	-0.095	0.159**	0.925	1.018
Skill discretion	-0.157*	0.139***	0.061	-0.310***	-0.111	0.273***	0.967**	0.994
Δ*R*^2 ^*attributable to level & work stress*	0.037*	0.083	0.033	0.150***	0.093***	0.121***	68.60^b^***	27.52^b^**

All analyses controlling for age, education, household responsibilities, and life events in past 6 months

a Incidence Rate Ratio from negative binomial or Poisson regression;

b Chi-square estimate of improvement in fit of the regression model due to employee level and work stress factors.

c Standardised Beta

* p < 0.05; ** p < 0.01; *** p < 0.001

For women, we found no effect of employee level on their measures of mental or physical health. However, employment level was associated with GP service use with those in middle management positions being less likely to have obtained GP care, compared with those at the executive level. Women's levels of mental health and well-being were better when they worked in a job that offered higher levels of skill discretion. Manageability of job demands impacted on all health measures considered while those who worked longer hours were more likely to have obtained GP services.

Finally, we explored the contributions of employment level and work stress factors in explaining participants' use of GP services, after including in the model SF-12 measures of mental and physical health that could also contribute to explaining such service use. Again, men and women were considered separately. Overall, controlling for mental and physical health in addition to sociodemographic measures had little impact on our findings. Men in lower employee categories continued to use significantly fewer GP services compared with their counterparts in executive positions while those with less job control and less job security again obtained more care. After controlling for mental and physical health, women in middle management levels again used fewer GP services compared with those at the executive level. Similarly, women with more manageable job demands and those working longer hours continued to be higher users of GP care.

## Discussion

This paper has reported on the associations between categories of employee levels, work stressors and mental health measures of 806 government employees aged between 40 and 44 who participated in the PATH Through Life Project being conducted in Canberra, Australia's national capital.

### Impact of employee level on work stressors, health and GP service use

Our first hypothesis, that government employees working at lower levels would report higher levels of work-related stress, is supported in the main by our research. Overall, participants at more senior levels reported that they had more control over aspects of their work, greater opportunities to do interesting work using a range of skills, more job security but also that they were subject to higher job demands. These results closely match those reported by Marmot and colleagues in their 1991 study of over 10,000 British civil servants aged between 35 and 55 [7].

We had hypothesized that those in lower level positions would have poorer physical and mental health compared with more senior staff. None of our results supports this hypothesis. Although both male and female participants in the higher grades reported better well-being as measured by higher positive affect, we found no mental health benefit associated with having a more senior position. Furthermore, for men, we found this result to be reversed when the analyses controlled for work stress factors. Men in lower level positions reported higher levels of positive affect and better mental health, as measured by the SF-12. Women's mental health and negative affect were not affected by their employee level both when this factor was considered alone and when work stress factors were taken into account. We were unable to replicate the finding by Marmot and colleagues that those in higher positions had significantly better physical health compared with their subordinates. This difference in our results might be explained by our having a smaller sample. However, it also indicates that while those working at lower levels are more likely to experience some types of work stressors, working at those lower levels, *per se*, is not automatically associated with poorer mental or physical health for Australian government employees. While these findings are unusual, they do align with some previous research undertaken in the UK showing that those working in lower grades had better mental health [18]. One possible interpretation of our results is that men and women of this age group who have continued to work at lower employee levels may be pursuing satisfying goals in other areas of their lives, for example, family, outside business, recreational pursuits. Of course, confirmation of such an interpretation would require more detailed information from participants concerning their working arrangements and life priorities.

We found government employee level affected men's use of GP services but the direction of this effect was the reverse of that hypothesised. After taking into account education, other personal measures and levels of work stress, men at lower levels of the public service used fewer GP services than their superiors. This aligns with the previous finding that men at lower level positions also had better mental health [18] and suggests that further research is needed on the health drawbacks for men of their rising through levels of government employment. We also found that women in middle-management levels were less likely to have obtained GP services compared with their more senior counterparts.

### The impact of work stress on health and GP service use

Work stress factors experienced at all levels of employment played a more significant role in affecting participants' health and well-being. For male and female participants, those whose job demands were more manageable had significantly better mental health. This measure of work stress was particularly important for women, for whom manageability of demands was significantly associated with all health measures. Correspondingly, level of skill discretion had a greater impact on men's mental health. This last finding concurs with a recent Australian study of government employees which reported that, for men, having interesting work was an important reason for their staying in the government sector whereas women saw their relative job security as the advantage of this form of employment [19].

Since previous research has linked job insecurity and poorer mental health [20,21], we expected similar findings in this current study. Women with less job security had less positive and more negative affect. Men with less job security, on the other hand, scored significantly worse on all six mental health measures. This finding, with that of the Australian study reported above, suggests that, while women place value on security, this and other work attributes may play a less important role in affecting their overall mental health, compared with their male counterparts. Previous studies have also reported sex differences concerning the mental health consequences of job insecurity [22]. Job security has also previously been found to affect the physical health of men more than women [23,24]. However, the one measure of physical health used in this study was not directly affected by job insecurity for men or women.

A number of work stress factors contributed significantly to explaining men's and women's use of GP care. Women who used more services had less job control while men were more likely to visit a GP if they reported less skill discretion, job control or felt more insecure about their job. Research reported in the 1980's also found that factory workers with higher job insecurity used more health services [25]. This finding in our study of government employees suggests that job insecurity has an important effect on men's, but not women's, self-assessment of their levels of health and well-being.

Women who worked longer hours used slightly more GP services and also had more manageable job demands. Men who worked fewer hours, however, obtained more GP care. These findings indicate that time spent at work is unlikely to have deterred women from addressing their health care needs. For men, on the other hand, working fewer hours per week might have given them needed, additional opportunity to set aside time for a GP visit. In Australia, many GP surgeries are open during standard working hours and offer only limited access outside of these times [26].

### Limitations

This study is limited by having access only to cross-sectional data, since the direction of relationships between psychological stress and work stressors cannot be confirmed. It may be that those with fewer symptoms of depression or anxiety, for example, also have more positive views about the current or potential benefits of their positions. A number of issues raised here may become clearer when data from the next wave of the PATH project are collected.

## Conclusions

These findings have implications for governments in their role as major employers. Large organisations in the public and private sectors inevitably develop hierarchical systems of employment as efficient mechanisms for allocating work functions and responsibilities. This study suggests that employees' health is less affected by their position within this structural hierarchy and is more associated with various work stressors that can be experienced by individuals across all levels of employment. For a large employer, reducing the impact of work stress on its workforce may be beneficial, not only for individual employees, but also for the productivity of the organisation as a whole. These findings also have implications specific to the Australia setting where GP services are provided and subsidised through the universal health insurance system, Medicare. This component of health care is the financial responsibility of the Australian Government, which is also the employer of the majority of participants in this current study. Initiatives aimed at reducing work stress experienced by government employees, and correspondingly, the numbers of GPs services obtained by that workforce, might prove to be a judicious use of Australian Government resources. Such potential benefits may well apply to other governments that have responsibility for funding health care services.

## Competing interests

The authors declare that they have no competing interests.

## Abbreviations

ACT Australian Capital Territory

APS Australian Public Service

GP General practitioner

IRR Incidence rate ratio

PANAS Positive and Negative Affect Scale

SF-12 12-item Short Form Health Survey

SPSS Statistical Package for Social Scientists

## Authors' contributions

RP contributed to the conception of this study, and was responsible for all analyses of data, interpretation of results and writing of successive drafts of the paper. AFJ was leader in designing and running the PATH Through Life Project, contributed to interpretation of findings and edited successive drafts of the paper. HC was co-leader in designing and running the PATH Through Life Project, contributed to interpretation of findings and edited successive drafts of the paper. DB, LS and RD contributed to the conception of the study and editing successive drafts of the paper. All authors read and approved the final version of the manuscript.

**Table 2:** Mean sociodemographic measures, work stress and working hours by government employee categories

	Government employee categories					
	Junior	Intermediate	Senior 1	Senior 2	Executive	*P*
	
Years of education	13.46	14.87	15.69	16.03	16.58	0.37
Number of life events in past 6 months	1.02	0.95	0.92	0.89	0.73	0.59
Level of household responsibility	9.90	9.80	9.66	9.57	8.42	0.01
Job control score*	2.91	3.12	3.25	3.29	3.35	<0.01
Manageable job demands score*	2.33	2.28	2.03	1.81	1.63	<0.01
Skill discretion score*	2.76	3.13	3.35	3.42	3.54	<0.01
Job security*	2.64	2.76	2.78	2.89	2.91	0.02
Number of hours worked per week	36.78	39.20	43.20	48.06	53.73	<0.01

* Individuals' responses were scored from 1 to 4. Higher scores were given to work circumstances in which the individual had more job control, more manageable job demands, higher skill discretion and greater job security.

## Pre-publication history

The pre-publication history for this paper can be accessed here:


